# Pre-Activated Granulocytes from an Autoimmune Uveitis Model Show Divergent Pathway Activation Profiles upon IL8 Stimulation In Vitro

**DOI:** 10.3390/ijms23179555

**Published:** 2022-08-23

**Authors:** Anne L. C. Hoffmann, Stefanie M. Hauck, Cornelia A. Deeg, Roxane L. Degroote

**Affiliations:** 1 Chair of Physiology, Department of Veterinary Sciences, LMU Munich, D-82152 Martinsried, Germany; 2 Research Unit Protein Science, Helmholtz Center Munich, German Research Center for Environmental Health, D-80939 Munich, Germany

**Keywords:** ERU, IL8, PMN, pre-activated granulocytes, extravasation

## Abstract

In the pathophysiology of autoimmune-mediated uveitis, granulocytes have emerged as possible disease mediators and were shown to be pre-activated in equine recurrent uveitis (ERU), a spontaneous disease model. We therefore used granulocytes from ERU horses to identify early molecular mechanisms involved in this dysregulated innate immune response. Primary granulocytes from healthy and ERU horses were stimulated with IL8, and cellular response was analyzed with differential proteomics, which revealed significant differences in protein abundance of 170 proteins in ERU. Subsequent ingenuity pathway analysis identified three activated canonical pathways “PKA signaling”, “PTEN signaling” and “leukocyte extravasation”. Clustered to the leukocyte extravasation pathway, we found the membrane-type GPI-anchored protease MMP25, which was increased in IL8 stimulated ERU granulocytes. These findings point to MMP25 as a possible regulator of granulocyte extravasation in uveitis and a role of this molecule in the impaired integrity of the blood-retina-barrier. In conclusion, our analyses show a clearly divergent reaction profile of pre-activated granulocytes upon IL8 stimulation and provide basic information for further in-depth studies on early granulocyte activation in non-infectious ocular diseases. This may be of interest for the development of new approaches in uveitis diagnostics and therapy. Raw data are available via ProteomeXchange with identifier PXD013648.

## 1. Introduction

Understanding the molecular mechanisms involved in divergent immune response in autoimmune uveitis is indispensable for deeper insights into disease pathogenesis, which in consequence may contribute to new approaches in diagnostics and therapy. Although a dysregulated T cell response is widely recognized as a key driver in disease pathogenesis, the involvement of innate immune cells has also become evident [1,2]. Early ocular influx of granulocytes was described in rodent models of experimental autoimmune uveitis [1,3,4,5,6,7] and has also been observed in ERU horses [8]. However, the specific role of these cells in onset and relapsing of disease is not completely understood so far.

Equine recurrent uveitis (ERU) is a spontaneously occurring, remitting-relapsing painful disease and a leading cause of blindness among horses worldwide [9,10]. In warmblood horses, ERU presents mostly as pan-uveitis or posterior uveitis with recurrent inflammation of choroid, retina and vitreous, and does not occur in combination with other symptoms in non-ocular tissue [11]. Besides its importance for veterinary medicine, it is also a valuable spontaneous model for non-infectious autoimmune-mediated recurrent pan-uveitis in humans, due to strong clinical and immunopathological similarities such as occurrence of shared autoantigens (cellular retinaldehyde-binding protein (CRALBP), interphotoreceptor retinoid-binding protein (IRBP), soluble antigen (S-Ag)) [12,13,14,15,16] and spontaneous onset of disease with remitting-relapsing character and unsolved etiology [17,18]. Concordant with recurrent autoimmune uveitis, ERU is demonstrably T-cell driven, but ocular infiltration by granulocytes has also been observed [8]. Previously, we could show that circulating granulocytes change their protein abundance repertoire in ERU [19,20,21]. While proteome changes of granulocytes with low density pointed to regulation of integrin signaling in ERU [19,20], normal-density granulocyte-derived proteins with increased abundance in ERU strongly associated to RAF/MAP kinase signaling and MHC-I antigen presentation, as well as neutrophil degranulation and impairment of blood-retina-barrier (BRB) integrity [21]. The enrichment of said pathways points to deviant functional properties of circulating granulocytes in ERU, suggesting that these cells are no longer in a resting state, but rather show a phenotype that is more readily activated, a feature also observed in other T cell driven diseases [22]. Therefore, we hypothesized that through this pre-activated phenotype, granulocytes might play a more prominent role in ERU pathogenesis than initially thought. Since we were especially interested in the behavior of these pre-activated granulocytes in an inflammatory environment, we analyzed their reaction profile to stimulation with interleukin 8 (IL8), a potent and specific chemoattractant for granulocytes [23]. With these studies, we aim to gain more insight into the early molecular mechanisms involved in dysregulated innate immune response in recurring uveitis and the role of granulocytes in T cell mediated ocular disease.

## 2. Results

### 2.1. Short Stimulation with IL8 Shows Divergent Reaction Profile in Granulocytes from Horses with Recurrent Uveitis

**Figure 1:**
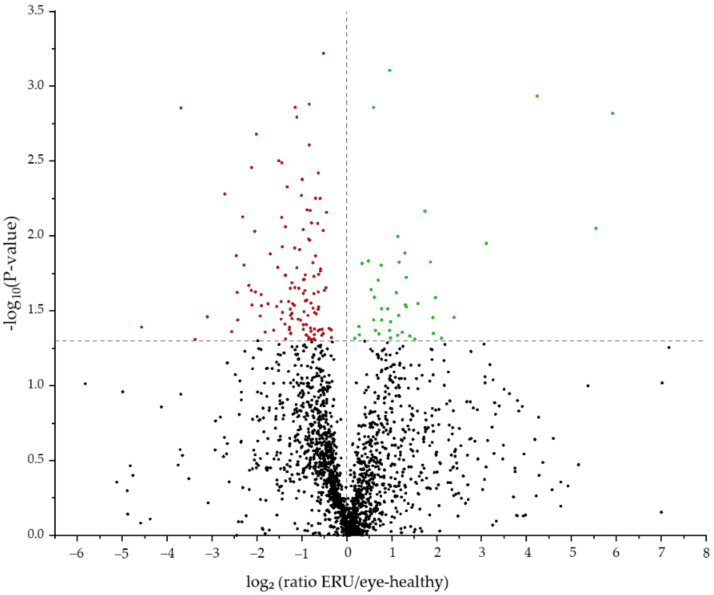
Volcano plot of the 2012 proteins detected by mass spectrometry post IL8 stimulation. The 170 differentially abundant candidates (*p* < 0.05) between controls and ERU cases are displayed above the grey line. More abundant proteins are displayed in green, and less abundant proteins are marked red.

With differential proteomics, we identified 2012 proteins in equine granulocytes (Figure 1, Table S1). Comparison of the proteomes of IL8-stimulated granulocytes revealed 170 proteins with significantly altered abundance between healthy controls and ERU cases (Table S1). Of these differentially expressed proteins, 126 were less abundant and 44 were more abundant in granulocytes of horses with ERU (Figure 1).

### 2.2. Canonical Pathways Protein Kinase A Signaling, PTEN Signaling and Leukocyte Extravasation Are Activated in Granulocytes of ERU Horses

**Figure 2:**
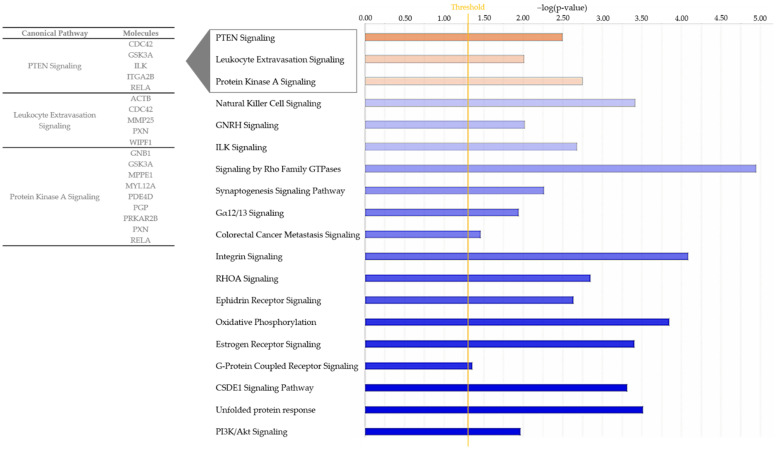
Bar chart showing all enriched pathways with a positive (orange) or negative (blue) z-score for the prediction of activation in ingenuity pathway analysis. The pathways shown are associated to proteins from IL8 stimulated ERU granulocytes. Color intensity correlates with z-score, while bar length indicates statistical significance. The orange line represents the p-value threshold. Gene names of differentially abundant proteins from our input dataset (*p* < 0.05) associating with the three activated pathways are shown.

**Figure 3:**
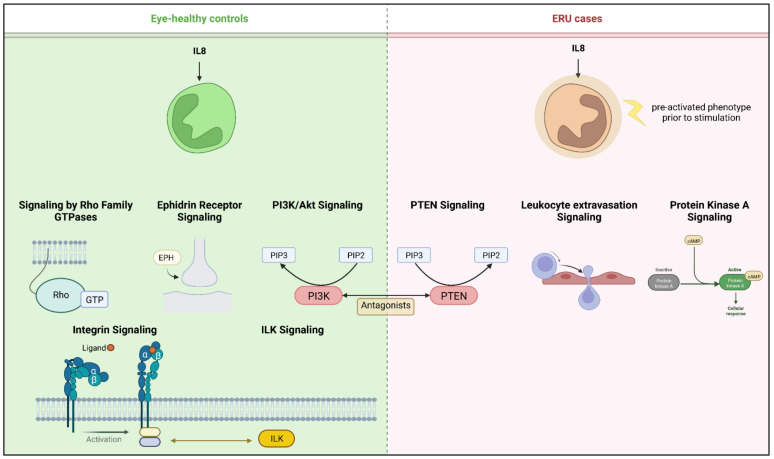
Overview of pathways with predicted activation after IL8 stimulation of equine granulocytes. Left panel shows canonical pathways activated in granulocytes from healthy horses; for better clarity, pathways from the same functional category were merged. Right panel shows respective activation in intrinsically pre-activated ERU granulocytes. Created with BioRender.com.

For interpretation of the detected protein abundance differences in a broader biological context, we subjected our dataset to ingenuity pathway analysis (IPA; Quiagen, Hilden, Germany), which revealed a total of 76 significantly enriched canonical pathways (Table S2). While 57 of these pathways either did not show a changed activity pattern between groups or did not allow activity pattern prediction (Table S2), we detected 16 canonical pathways, which were predicted to be inhibited in ERU samples (Figure 2, Table S2). Among these, the strongest inhibition was predicted for “phosphatidylinositol 3-kinase/Aktin (PI3K/Akt) signaling” (Figure 2, Table S2). In contrast, the three pathways “protein kinase A (PKA) signaling”, “phosphatase and tensin homologue (PTEN) signaling” and “leukocyte extravasation” showed a clearly predicted activation in IL8 stimulated granulocytes from ERU cases compared to eye-healthy controls (Figure 2 and Figure 3, Table S2).

### 2.3. Activated Pathways in ERU Associate with Processes in Cell Motility and Migration

All activated pathways identified with IPA associated with processes in cell motility and migration. This is reflected in the proteins allocated to these pathways, which are mainly associated with cytoskeleton organization, cell adhesion and cell migration. In detail, nine differentially expressed proteins from our dataset clustered to protein kinase A (PKA) signaling: G protein subunit beta 1 (GNB1), glycogen synthase kinase 3 alpha (GSK3A), metallophosphoesterase 1 (MPPE1), myosin light chain 12A (MYL12A), phosphodiesterase 4D (PDE4D), phosphoglycolate phosphatase (PGP), protein kinase cAMP-dependent type II regulatory subunit beta (PRKAR2B), paxillin (PXN) and RELA proto-oncogene, NF-kB subunit (RELA) (Table S2, Figure 2). Five proteins were allocated to the PTEN signaling pathway, namely cell division cycle 42 (CDC42), glycogen synthase kinase 3 alpha (GSK3A), integrin-linked kinase (ILK), integrin subunit alpha 2b (ITGA2B) and RELA proto-oncogene, NF-kB subunit (RELA) (Table S2, Figure 2).

Since leukocyte extravasation is a crucial step in the pathogenesis of ERU, we were especially interested in the proteins allocated to this pathway (Table S2, Figure 2). Five proteins from our dataset were mapped to this pathway, of which four were associated with actin cytoskeleton organization and signaling, namely actin beta (ACTB) and cell division cycle 42 (CDC42) (both upregulated in ERU) as well as paxillin (PXN) and “WASWASL interacting protein family member 1” (WIPF1) (both downregulated in ERU) (Table S1). The fifth protein allocated to the leukocyte extravasation pathway was the plasma-membrane-bound matrix metallopeptidase 25 (MMP25), which showed the highest fold change among all of the five allocated proteins (2.5-fold, Figure 2, Table S1).

## 3. Discussion

The participation of granulocytes in non-infectious recurrent uveitis pathogenesis has been shown in rodent uveitis models [1,2,3,6]. However, the exact role of these cells and their timing in and impact on ERU pathogenesis has not been completely understood to date. On the one hand, transmigration of granulocytes over the BRB into the eye may occur as a secondary phenomenon caused by cytokine release from infiltrated lymphocytes. On the other hand, early influx of granulocytes might be an initiating factor for autoreactive behavior of adaptive immune cells. Horses and humans share a wide range of similarities regarding the composition and function of their immune system [24,25] and equine immune cells can be used as valuable tools for studying human diseases [18,26,27,28]. In particular, the availability of equine ocular tissue makes the horse an attractive translational model for studies on non-infectious autoimmune-mediated recurrent pan-uveitis. We could previously show in said spontaneous recurrent uveitis model, that granulocytes display an activated phenotype per se, even between uveitic attacks in quiescent stage of disease [21], which points to the possibility of an active rather than a bystander role in disease pathogenesis. In this study, we now detected a distinct pattern of differentially regulated proteins and associated pathways in IL8 stimulated granulocytes of ERU cases, which shows that granulocytes with a pre-activated phenotype in ERU horses react differently than their healthy counterpart.

Since IL8 is a chemokine, which induces chemotaxis and NETosis in primary human granulocytes [29], we also expected to find proteins associated with these functions in horse granulocytes. In this context, we were especially interested in the response patterns of the intrinsically pre-activated granulocytes from ERU horses after stimulation with IL8 in vitro. Although our discovery proteomics experiment included 30 samples and stimulation of cells with a total of three different stimuli (https://www.ebi.ac.uk/pride/archive, dataset identifier PXD013648 (accessed on 15 January 2020)) [21,23], we chose to specifically analyze the data from IL8 stimulation because we wanted to explore possible differences in migration from the peripheral bloodstream over the BRB into the eye. Unlike the other two stimulants used, namely phorbol 12-myristate 13-acetate (PMA), which mainly induces exocytosis, ROS release and NET formation via protein-kinase-C [30], and lipopolysaccharide (LPS), which triggers similar responses by binding to TLR4 on neutrophils [31], IL8 has s been proven to be a strong and specific chemoattractant for neutrophils via CXCR1 and CXCR2 receptors (reviewed in [32]), especially for migration over endothelial and epithelial barriers [33,34]. In the presence of IL8, neutrophils show a flattened and elongated morphology, which is characteristic for the migration process [35]. Increased levels of IL8 have been described in blood and vitreous of uveitis patients with Behçet’s disease [36,37] and IL8 was significantly increased in tears of children with Juvenile Idiopathic Arthritis-associated Uveitis during an episode of active uveitis, but not in quiescent stage [38]. Moreover, IL8 and the upregulation of its receptors have been linked to disease severity in several rodent models for different types of uveitides [39,40,41] and the IL8 homologue in mice, CXCL1, has been suggested to take part in neutrophil infiltration in RBP-3 induced experimental autoimmune uveitis (EAU) [1]. Interestingly, the neutrophil infiltration peak in said mouse model coincides with the highest relative number of infiltrated Th17 cells, suggesting a possible regulatory effect of IL17 secreting T cells on neutrophil mobilization and migration into the eye during uveitis [1]. In recurrent autoimmune uveitis and EAU, both Th1 and Th17 immune response are proven to drive disease [42]. In ERU, on the other hand, increased IFNγ and decreased IL10 expression levels in CD4^+^ T cells [43] point to an activated Th1 response, whereas the exact role of Th17 cells has not been determined to date. Regulation of Th17 immune response has been suggested to take place through a presumable IL33/IL31 cytokine axis, especially in inflammatory and allergic disorders [44,45]. But, although IL33 and IL31 are increased in serum of uveitis patients with Behçet’s disease (reviewed in [46]) and IL33 has been described to play a role in the course of EAU [47], the exact impact of the IL33/IL31 interplay on neutrophils in autoimmune uveitis is still unknown and merits further investigations.

Despite the possibility of in vivo granulocyte priming through undetected systemic inflammation prior to blood withdrawal, an impact on the analysis results presented here is unlikely since the ERU horses used in our study underwent daily clinical health assessment and did not show any signs of active inflammation. Using discovery proteomics paired with ingenuity pathway analysis, we identified distinct differences in predicted pathway activation as a reaction to IL8 stimulation between granulocytes from healthy horses and ERU cases (Figure 2 and Figure 3, Table S2). Among the inhibited pathways in ERU granulocytes, we identified the PI3K/Akt signaling pathway (Figure 2 and Figure 3, Table S2), which is involved in neutrophil cytoskeleton dynamics [48,49]. Inhibition of the PI3K/Akt signaling pathway in human and mouse neutrophils could show that, although it plays a role in polarization and movement of these cells, it is not a major player in signal generation for directed movement [48]. The predicted inhibition of this pathway in stimulated ERU granulocytes may therefore indicate downstream suppression of random chemokinesis in favor of directional chemotaxis. This is supported by the predicted activation of PKA-, PTEN- and leukocyte extravasation signaling pathways in these cells, which are associated with migration and chemotaxis, thus directed movement [50,51,52] (Figure 2 and Figure 3, Table S2). The role of activated PKA signaling in equine granulocytes has not been investigated in the context of recurrent autoimmune uveitis so far; however, the majority of studies on human or rodent neutrophils report a complex regulatory mechanism of this pathway in immune cell function (reviewed in [53]). Whether this is similar in equine neutrophils is still an elusive question that merits further investigations. PTEN, which was also activated in ERU granulocytes (Figure 2 and Figure 3, Table S2), catalyzes the dephosphorylation of phosphoinositol-(3,4,5)-triphosphat (PIP3) to phosphoinositol-(4,5)-diphosphat (PIP2) and is therefore a direct antagonist of PI3K, which phosphorylates PIP2 to PIP3 (reviewed in [54]). Interestingly, in our analysis, PI3K/Akt signaling was among the inhibited pathways in IL8 stimulated ERU granulocytes, while the PTEN signaling pathway was predicted to be activated, reflecting this reverse effect. To our knowledge we are the first to describe an activated PTEN Signaling in ERU, though it is in accord with our results in previous studies, showing that PI3K signaling is activated in granulocytes of eye-healthy controls after IL8 stimulation [23]. Overexpression of PTEN was linked to an increased NET formation in human promyelocytic leukemia (HL-60) differentiated neutrophil-like cells [55], a process that is also more readily activated in neutrophils of ERU horses [56]. Although PTEN signaling serves complex functions in neutrophil chemotaxis, cell motility and polarity ([51], reviewed in [54]), association with autoimmunity and autoimmune disease is mainly through inhibition of this pathway [57,58,59]. Most of the studies describing the involvement of PTEN in autoimmunity manly focus on T cells or B cells [60], while the role of PTEN signaling in neutrophils in terms of autoimmunity is poorly examined so far. To obtain deeper insight into the possible function of PTEN signaling in granulocytes and to validate our hypothesis of its association with recurrent autoimmune uveitis, further studies with a large validation cohort are necessary.

When immune cells are activated in the periphery and leave the bloodstream to migrate into the eye, they cause damage to intraocular structures [14,61,62]. There are several theories on how peripheral immune cells manage to overcome the BRB, which is designed to maintain ocular immune privilege. For example, loss of barrier integrity might originate in the cells forming the barrier itself. On the other hand, it may occur as a secondary effect in response to the recruitment of peripheral immune cells. Whatever the cause, aktin cytoskeleton dynamics is a key player driving these mechanisms and a prerequisite for leukocyte extravasation [63]. Therefore, the predicted activation of proteins of the “leukocyte extravasation pathway” in our analysis of ERU granulocytes after IL8 stimulation might indicate changes in the cellular function of granulocytes in ERU. Since said pathway was specifically activated in ERU granulocytes and not in the healthy controls (Figure 2 and Figure 3, Table S2), this might point to differences needed for successful migration over the BRB, which needs to be addressed in more detail in future studies. Matrix metalloproteinase 25 (MMP25) was among the changed proteins in ERU granulocytes clustering to this pathway (Figure 2/Table S2). MMP25 is a membrane-type GPI anchored protease, which belongs to the matrix-metalloproteinase family [64,65] and plays a role in extracellular matrix remodeling and proteolysis, as well as inflammatory responses such as trans-endothelial migration of neutrophils to inflammatory sites [66,67]. Other MMPs have previously been associated with ERU pathogenesis through changed expression levels in the equine retina and in infiltrated Th1 cells [68]; however, MMP25 has not been investigated in recurrent autoimmune uveitis so far. In other autoimmune-mediated diseases, it has been described as a promising drug target due to its role in activated proteolytic pathways [69]. Moreover, MMPs compromise blood-brain-barrier integrity through proteolyzation of basement membrane and tight junction proteins in autoimmune-mediated disorders such as multiple sclerosis, allowing infiltration of peripheral immune cells [70,71]. The increased abundance of MMP25 in our dataset might therefore indicate a similar effect on the BRB in recurrent autoimmune uveitis; however, future migration and motility studies are needed to confirm our hypothesis and shed more light on the role of this molecule in ERU pathogenesis.

Although we used biological replicates from outbred animals kept in normal surroundings, rather than rodents from one breed kept in laboratory facilities or only technical replicates from the same sample, a limitation of our study is the overall sample size: we used granulocytes from three healthy horses and three horses with ERU for our analyses. Since we aimed at a hypothesis-generating approach, focusing on the depth of proteome coverage rather than the cohort size, the number of samples used in our study is of standard size for discovery proteomics. With stringent mass spectrometry settings, the smaller sample size in this initial discovery stage and individual variance allow at least hypothesis building. Nevertheless, the statistical power of our hypotheses and conclusions needs to be strengthened through verification and validation experiments with significantly increased sample size. To improve the robustness of our findings, further analyses are currently being established, and will be conducted in future experiments.

In summary, discovery proteomics paired with ingenuity pathway analysis showed, that pre-activated granulocytes from ERU cases exhibit changes in their proteome, which indicate an aberrant response profile to in vitro stimulation with the chemokine IL8. Since granulocytes show said pre-activated phenotype in ERU, we hypothesize that these cells may be changed is a way that promotes immune dysregulation in vivo, suggesting a more active role in recurrent autoimmune uveitis pathogenesis than expected. Because the spontaneously occurring disease in the horse is a valuable model for non-infectious recurrent autoimmune uveitis in humans, these findings may serve as a basis for more in-depth studies of early granulocyte activation in non-infectious ocular diseases and may help to gain more insights in disease pathogenesis and the interplay of granulocytes and T cells in autoimmune diseases.

## 4. Materials and Methods

### 4.1. Sample Processing

Primary granulocytes from heparinized (50 I.U./mL blood, Ratiopharm, Ulm, Germany) venous whole blood of three healthy horses and three ERU cases were used in this study. The healthy horses are owned by the Equine Clinic at Ludwig-Maximilians-University Munich. Horses with ERU were patients awaiting therapeutic procedure. ERU was diagnosed by experienced clinicians from the Equine Clinic at Ludwig-Maximilians-University Munich and was based on typical clinical signs of uveitis along with a documented history of multiple episodes of inflammation of the affected eye [72]. In all three horses only one eye was affected and showed a classical ERU, characterized as panuveitis during the uveitic attack. At the time of blood withdrawal, ERU horses were in quiescent stage of disease. At this point two horses showed minor deposits at the back of the lens and minor impurities in the vitreous. One horse also had a ventral posterior synechia. The third horse showed subcapsular vesicular cataract of the lens. All horses were clinically examined and showed no impairment of their health status except for the alterations in the eye linked to ERU diagnosis. No experimental animals were used in this study. Collection of blood was permitted by the local authority (Regierung von Oberbayern, Permit number: ROB-55.2Vet-2532.Vet_03-17-88).

After rough sedimentation of erythrocytes, granulocytes were isolated from plasma by density gradient centrifugation (room temperature, 350× *g*, 25 min, brake off) with Ficoll-Paque PLUS separating solution (density 1.077 g/mL; Cytiva Life Sciences, Freiburg, Germany). The resulting interphase, containing PBMC, was discarded, cells at the bottom of the tube were carefully washed (4 °C, 400× *g*, 10 min) in cold PBS (DPBS devoid of CaCl_2_ and MgCl_2_; Gibco/Thermo Fisher Scientific, Darmstadt, Germany) and remaining erythrocytes were removed by 30 s sodium chloride (0.2% NaCl) lysis. Isotonicity of samples was restored through addition of equal parts 1.6% NaCl. Granulocytes were then washed (4 °C, 400× *g*, 10 min) and resuspended in PBS with 0.2% glucose.

From each animal used in the experiment, we prepared five aliquot portions of 6 × 10^5^ cells/500 µL, resulting in a total of 30 samples. Per animal, one aliquot was left as is, one was used as medium control (=untreated but incubated under the same conditions as stimulated cells) and the remaining three were stimulated with different stimulants (PMA, LPS, IL8) for 30 min in a CO_2_ incubator (37 °C, 5% CO_2_). For the analysis described in this manuscript, we used the data from granulocytes stimulated with recombinant equine interleukin-8 (IL8; Kingfisher Biotec; 1ng/mL). The data from granulocyte stimulation with the other two stimulants have been published elsewhere [23]. After the 30 min incubation, the volume of each aliquot was adjusted to 1ml by adding PBS with 0.2% Glucose. Cells were then pelleted (4 °C, 2300× *g*, 10 min) and stored at −20 °C. Prior to mass spectrometric analysis, granulocytes were thawed and lysed in urea buffer (8M urea in 0.1M Tris/HCl pH 8.5), and protein concentration was determined with Bradford protein assay [73].

### 4.2. Mass Spectrometric Analysis

All 30 samples were analyzed in one setting. From each sample, 10 µg total protein was digested with LysC and trypsin by filter-aided sample preparation (FASP) as previously described [74]. Acidified eluted peptides were analyzed in a data-dependent mode on a QExactive HF mass spectrometer (Thermo Fisher Scientific) online coupled to a UItimate 3000 RSLC nano-HPLC (Dionex). Samples were automatically injected and loaded onto the C18 trap cartridge and after 5 min eluted and separated on the C18 analytical column (nanoEase MZ HSS T3, 100 Å, 1.8 µm, 75 µm × 250 mm; Waters) by a 95 min non-linear acetonitrile gradient at a flow rate of 250 nL/min. MS spectra were recorded at a resolution of 60,000 with an automatic gain control (AGC) target of 3e^6^ and a maximum injection time of 30 ms from 300 to 1500 m/z. From the MS scan, the 10 most abundant peptide ions were selected for fragmentation via HCD with a normalized collision energy of 27, an isolation window of 1.6 m/z, and a dynamic exclusion of 30 s. MS/MS spectra were recorded at a resolution of 15,000 with a AGC target of 1e^5^ and a maximum injection time of 50 ms. Unassigned charges, and charges of +1 and >+8 were excluded from precursor selection.

### 4.3. Data Processing and Label-Free Quantification

Label-free quantitative analysis was performed using Progenesis QI software (version 2.5, Nonlinear Dynamics, Waters, Newcastle upon Tyne, UK) as described [75,76]. Briefly, raw MS files were imported, followed by automatic peak picking and retention time alignment and normalization of total peak intensities across all samples to minimize loading differences. All MS/MS spectra were exported from Progenesis QI software as Mascot generic files (mgf) and searched against Ensembl Horse protein database (version 3.0,) for peptide identification with Mascot (version 2.5.1). Search parameters used were 10 ppm peptide mass tolerance, 20 mmu fragment mass tolerance, one missed cleavage allowed, carbamidomethylation as fixed modification and methionine oxidation as well as deamidation of asparagine and glutamine as variable modifications. Mascot integrated decoy database search was set to a false discovery rate (FDR) of 1% when searching was performed on the concatenated mgf files with a percolator ion score cut-off of 13 and an appropriate significance threshold p. Identifications were re-imported into Progenesis QI and redundancies grouped following the rules of parsimony.

### 4.4. Data Analysis

Differential protein abundance of IL8 stimulated healthy and ERU granulocytes was determined by comparison of the mean normalized peptide abundance from the extracted ion chromatograms. Proteins abundance differences were considered significant at *p* < 0.05. Bioinformatic analysis was performed on human orthologues (rarely, on mouse orthologues, if human orthologue gene name was not available) of gene names from significant, differentially expressed equine proteins with Ingenuity Pathway Analysis (IPA; Qiagen, Hilden, Germany, https://digitalinsights.qiagen.com/ (accessed on 20 May 2022)). Z-score describes prediction of activation or inhibition of respective pathway, significance threshold was set to –log(*p*-value) > 1.3. IPA analyses overrepresentation of proteins from data input in canonical pathways deposited in the IPA library, as previously described [77]. This allows insight on possible physiological effects of upstream molecules on these proteins and allocation to downstream pathways. Analysis was performed based on the abundance ratio p-value of the stimulated samples. Volcano plot was visualized with OriginPro 2020 software (Additive, Friedrichsdorf, Germany).

## Data Availability

The raw mass spectrometry proteomics data of the entire discovery proteomics dataset have been deposited to the ProteomeXchange Consortium via the PRIDE [78] partner repository (https://www.ebi.ac.uk/pride/archive, accessed on 15 January 2020) with the dataset identifier PXD013648.

## Supplementary Materials

The following supporting information can be downloaded at: https://www.mdpi.com/article/10.3390/ijms23179555/s1.

## References

[1] Kerr E.C., Raveney B.J., Copland D.A., Dick A.D., Nicholson L.B. (2008). Analysis of retinal cellular infiltrate in experimental autoimmune uveoretinitis reveals multiple regulatory cell populations. J. Autoimmun..

[2] Kerr E.C., Copland D.A., Dick A.D., Nicholson L.B. (2008). The dynamics of leukocyte infiltration in experimental autoimmune uveoretinitis. Prog. Retin. Eye Res..

[3] Pepple K.L., Wilson L., Van Gelder R.N. (2018). Comparison of Aqueous and Vitreous Lymphocyte Populations From Two Rat Models of Experimental Uveitis. Investig. Ophthalmol. Vis. Sci..

[4] Jones L.S., Rizzo L.V., Agarwal R.K., Tarrant T.K., Chan C.C., Wiggert B., Caspi R.R. (1997). IFN-gamma-deficient mice develop experimental autoimmune uveitis in the context of a deviant effector response. J. Immunol..

[5] Kim S.J., Zhang M., Vistica B.P., Chan C.C., Shen D.F., Wawrousek E.F., Gery I. (2002). Induction of ocular inflammation by T-helper lymphocytes type 2. Investig. Ophthalmol. Vis. Sci.

[6] Su S.B., Grajewski R.S., Luger D., Agarwal R.K., Silver P.B., Tang J., Tuo J., Chan C.C., Caspi R.R. (2007). Altered chemokine profile associated with exacerbated autoimmune pathology under conditions of genetic interferon-gamma deficiency. Investig. Ophthalmol. Vis. Sci..

[7] Caspi R.R., Chan C.C., Fujino Y., Najafian F., Grover S., Hansen C.T., Wilder R.L. (1993). Recruitment of antigen-nonspecific cells plays a pivotal role in the pathogenesis of a T cell-mediated organ-specific autoimmune disease, experimental autoimmune uveoretinitis. J. Neuroimmunol..

[8] Deeg C.A., Kaspers B., Gerhards H., Thurau S.R., Wollanke B., Wildner G. (2001). Immune responses to retinal autoantigens and peptides in equine recurrent uveitis. Investig. Ophthalmol. Vis. Sci..

[9] Gilger B.C., Michau T.M. (2004). Equine recurrent uveitis: New methods of management. Vet. Clin. N. Am. Equine Pract..

[10] Allbaugh R.A. (2016). Equine recurrent uveitis: A review of clinical assessment and management. Equine Vet. Educ..

[11] Gerding J.C., Gilger B.C. (2016). Prognosis and impact of equine recurrent uveitis. Equine Vet. J..

[12] Deeg C.A., Raith A.J., Amann B., Crabb J.W., Thurau S.R., Hauck S.M., Ueffing M., Wildner G., Stangassinger M. (2007). CRALBP is a highly prevalent autoantigen for human autoimmune uveitis. Clin. Dev. Immunol..

[13] Deeg C.A., Pompetzki D., Raith A.J., Hauck S.M., Amann B., Suppmann S., Goebel T.W., Olazabal U., Gerhards H., Reese S. (2006). Identification and functional validation of novel autoantigens in equine uveitis. Mol. Cell Proteom..

[14] Deeg C.A., Thurau S.R., Gerhards H., Ehrenhofer M., Wildner G., Kaspers B. (2002). Uveitis in horses induced by interphotoreceptor retinoid-binding protein is similar to the spontaneous disease. Eur. J. Immunol..

[15] Deeg C.A., Reese S., Gerhards H., Wildner G., Kaspers B. (2004). The uveitogenic potential of retinal S-antigen in horses. Investig. Ophthalmol. Vis. Sci..

[16] De Smet M.D., Bitar G., Roberge F.G., Gery I., Nussenblatt R.B. (1993). Human S-antigen: Presence of multiple immunogenic and immunopathogenic sites in the Lewis rat. J. Autoimmun..

[17] Malalana F., Stylianides A., McGowan C. (2015). Equine recurrent uveitis: Human and equine perspectives. Vet. J..

[18] Deeg C.A., Hauck S.M., Amann B., Pompetzki D., Altmann F., Raith A., Schmalzl T., Stangassinger M., Ueffing M. (2008). Equine recurrent uveitis—A spontaneous horse model of uveitis. Ophthalmic Res..

[19] Degroote R.L., Hauck S.M., Kremmer E., Amann B., Ueffing M., Deeg C.A. (2012). Altered expression of talin 1 in peripheral immune cells points to a significant role of the innate immune system in spontaneous autoimmune uveitis. J. Proteom..

[20] Degroote R.L., Hauck S.M., Treutlein G., Amann B., Frohlich K.J., Kremmer E., Merl J., Stangassinger M., Ueffing M., Deeg C.A. (2013). Expression Changes and Novel Interaction Partners of Talin 1 in Effector Cells of Autoimmune Uveitis. J. Proteome Res..

[21] Weigand M., Hauck S.M., Deeg C.A., Degroote R.L. (2020). Deviant proteome profile of equine granulocytes associates to latent activation status in organ specific autoimmune disease. J. Proteom..

[22] Naegele M., Tillack K., Reinhardt S., Schippling S., Martin R., Sospedra M. (2012). Neutrophils in multiple sclerosis are characterized by a primed phenotype. J. Neuroimmunol..

[23] Degroote R.L., Weigand M., Hauck S.M., Deeg C.A. (2019). IL8 and PMA Trigger the Regulation of Different Biological Processes in Granulocyte Activation. Front. Immunol..

[24] Horohov D.W. (2015). The equine immune responses to infectious and allergic disease: A model for humans?. Mol. Immunol..

[25] Zschaler J., Schlorke D., Arnhold J. (2014). Differences in innate immune response between man and mouse. Crit. Rev. Immunol..

[26] Bright L.A., Dittmar W., Nanduri B., McCarthy F.M., Mujahid N., Costa L.R., Burgess S.C., Swiderski C.E. (2019). Modeling the pasture-associated severe equine asthma bronchoalveolar lavage fluid proteome identifies molecular events mediating neutrophilic airway inflammation. Vet. Med..

[27] Vargas A., Boivin R., Cano P., Murcia Y., Bazin I., Lavoie J.P. (2017). Neutrophil extracellular traps are downregulated by glucocorticosteroids in lungs in an equine model of asthma. Respir. Res..

[28] Busch M., Wefelmeyer K.L., Walscheid K., Rothaus K., Bauer D., Deeg C.A., Degroote R.L., Ackermann D., Konig S., Thanos S. (2019). Identification of Ocular Autoantigens Associated With Juvenile Idiopathic Arthritis-Associated Uveitis. Front. Immunol..

[29] Bernhard S., Hug S., Stratmann A.E.P., Erber M., Vidoni L., Knapp C.L., Thomass B.D., Fauler M., Nilsson B., Nilsson Ekdahl K. (2021). Interleukin 8 Elicits Rapid Physiological Changes in Neutrophils That Are Altered by Inflammatory Conditions. J. Innate Immun..

[30] Saito T., Takahashi H., Doken H., Koyama H., Aratani Y. (2005). Phorbol myristate acetate induces neutrophil death through activation of p38 mitogen-activated protein kinase that requires endogenous reactive oxygen species other than HOCl. Biosci. Biotechnol. Biochem..

[31] Yipp B.G., Kim J.H., Lima R., Zbytnuik L.D., Petri B., Swanlund N., Ho M., Szeto V.G., Tak T., Koenderman L. (2017). The Lung is a Host Defense Niche for Immediate Neutrophil-Mediated Vascular Protection. Sci. Immunol..

[32] Metzemaekers M., Gouwy M., Proost P. (2020). Neutrophil chemoattractant receptors in health and disease: Double-edged swords. Cell. Mol. Immunol..

[33] Smart S.J., Casale T.B. (1993). Interleukin-8-induced transcellular neutrophil migration is facilitated by endothelial and pulmonary epithelial cells. Am. J. Respir. Cell Mol. Biol..

[34] Pieper C., Pieloch P., Galla H.J. (2013). Pericytes support neutrophil transmigration via interleukin-8 across a porcine co-culture model of the blood-brain barrier. Brain Res..

[35] Meyvantsson I., Vu E., Lamers C., Echeverria D., Worzella T., Echeverria V., Skoien A., Hayes S. (2011). Image-based analysis of primary human neutrophil chemotaxis in an automated direct-viewing assay. J. Immunol. Methods.

[36] Simsek M., Cakar Ozdal P., Akbiyik F., Citirik M., Berker N., Ozdamar Erol Y., Yilmazbas P. (2019). Aqueous humor IL-8, IL-10, and VEGF levels in Fuchs’ uveitis syndrome and Behcet’s uveitis. Int. Ophthalmol..

[37] Freire Ade L., Bertolo M.B., de Pinho A.J., Samara A.M., Fernandes S.R. (2004). Increased serum levels of interleukin-8 in polyarteritis nodosa and Behcet’s disease. Clin. Rheumatol..

[38] Angeles-Han S.T., Utz V.M., Thornton S., Schulert G., Rodriguez-Smith J., Kauffman A., Sproles A., Mwase N., Hennard T., Grom A. (2021). S100 proteins, cytokines, and chemokines as tear biomarkers in children with juvenile idiopathic arthritis-associated uveitis. Ocul. Immunol. Inflamm..

[39] Fang I.M., Yang C.H., Lin C.P., Yang C.M., Chen M.S. (2004). Expression of chemokine and receptors in Lewis rats with experimental autoimmune anterior uveitis. Exp. Eye Res..

[40] Brito B.E., O’Rourke L.M., Pan Y., Huang X., Park J.M., Zamora D., Cook D.N., Planck S.R., Rosenbaum J.T. (1999). Murine endotoxin-induced uveitis, but not immune complex-induced uveitis, is dependent on the IL-8 receptor homolog. Curr. Eye Res..

[41] Mo J.S., Matsukawa A., Ohkawara S., Yoshinaga M. (1999). Role and regulation of IL-8 and MCP-1 in LPS-induced uveitis in rabbits. Exp. Eye Res..

[42] Luger D., Silver P.B., Tang J., Cua D., Chen Z., Iwakura Y., Bowman E.P., Sgambellone N.M., Chan C.C., Caspi R.R. (2008). Either a Th17 or a Th1 effector response can drive autoimmunity: Conditions of disease induction affect dominant effector category. J. Exp. Med..

[43] Saldinger L.K., Nelson S.G., Bellone R.R., Lassaline M., Mack M., Walker N.J., Borjesson D.L. (2020). Horses with equine recurrent uveitis have an activated CD4+ T-cell phenotype that can be modulated by mesenchymal stem cells in vitro. Vet. Ophthalmol..

[44] Vocca L., Di Sano C., Uasuf C.G., Sala A., Riccobono L., Gangemi S., Albano G.D., Bonanno A., Gagliardo R., Profita M. (2015). IL-33/ST2 axis controls Th2/IL-31 and Th17 immune response in allergic airway diseases. Immunobiology.

[45] Di Salvo E., Ventura-Spagnolo E., Casciaro M., Navarra M., Gangemi S. (2018). IL-33/IL-31 Axis: A Potential Inflammatory Pathway. Mediators Inflamm..

[46] Murdaca G., Greco M., Tonacci A., Negrini S., Borro M., Puppo F., Gangemi S. (2019). IL-33/IL-31 Axis in Immune-Mediated and Allergic Diseases. Int. J. Mol. Sci..

[47] Barbour M., Allan D., Xu H., Pei C., Chen M., Niedbala W., Fukada S.Y., Besnard A.G., Alves-Filho J.C., Tong X. (2014). IL-33 attenuates the development of experimental autoimmune uveitis. Eur. J. Immunol..

[48] Ferguson G.J., Milne L., Kulkarni S., Sasaki T., Walker S., Andrews S., Crabbe T., Finan P., Jones G., Jackson S. (2007). PI(3)Kgamma has an important context-dependent role in neutrophil chemokinesis. Nat. Cell Biol..

[49] Feng S., Zhou L., Zhang Y., Lu S., Long M. (2018). Mechanochemical modeling of neutrophil migration based on four signaling layers, integrin dynamics, and substrate stiffness. Biomech Model. Mechanobiol..

[50] Jones S.L., Sharief Y. (2005). Asymmetrical protein kinase A activity establishes neutrophil cytoskeletal polarity and enables chemotaxis. J. Leukoc. Biol..

[51] Li Y., Jin Y., Liu B., Lu D., Zhu M., Jin Y., McNutt M.A., Yin Y. (2019). PTENalpha promotes neutrophil chemotaxis through regulation of cell deformability. Blood.

[52] McMinn P.H., Hind L.E., Huttenlocher A., Beebe D.J. (2019). Neutrophil trafficking on-a-chip: An in vitro, organotypic model for investigating neutrophil priming, extravasation, and migration with spatiotemporal control. Lab. Chip.

[53] Howe A.K. (2004). Regulation of actin-based cell migration by cAMP/PKA. Biochim. Biophys. Acta.

[54] Taylor H., Laurence A.D.J., Uhlig H.H. (2019). The Role of PTEN in Innate and Adaptive Immunity. Cold Spring Harb. Perspect. Med..

[55] Teimourian S., Moghanloo E. (2015). Role of PTEN in neutrophil extracellular trap formation. Mol. Immunol..

[56] Fingerhut L., Ohnesorge B., von Borstel M., Schumski A., Strutzberg-Minder K., Morgelin M., Deeg C.A., Haagsman H.P., Beineke A., von Kockritz-Blickwede M. (2019). Neutrophil Extracellular Traps in the Pathogenesis of Equine Recurrent Uveitis (ERU). Cells.

[57] Wu X.N., Ye Y.X., Niu J.W., Li Y., Li X., You X., Chen H., Zhao L.D., Zeng X.F., Zhang F.C. (2014). Defective PTEN regulation contributes to B cell hyperresponsiveness in systemic lupus erythematosus. Sci. Transl. Med..

[58] Zare-Chahoki A., Ahmadi-Zeidabadi M., Azadarmaki S., Ghorbani S., Noorbakhsh F. (2021). Inflammation in an Animal Model of Multiple Sclerosis Leads to MicroRNA-25-3p Dysregulation Associated with Inhibition of Pten and Klf4. Iran. J. Allergy Asthma Immunol..

[59] Bluml S., Sahin E., Saferding V., Goncalves-Alves E., Hainzl E., Niederreiter B., Hladik A., Lohmeyer T., Brunner J.S., Bonelli M. (2015). Phosphatase and tensin homolog (PTEN) in antigen-presenting cells controls Th17-mediated autoimmune arthritis. Arthritis Res. Ther..

[60] Huynh A., Turka L.A. (2013). Control of T cell tolerance by phosphatase and tensin homolog. Ann. N. Y. Acad. Sci..

[61] Xu H., Manivannan A., Jiang H.R., Liversidge J., Sharp P.F., Forrester J.V., Crane I.J. (2004). Recruitment of IFN-gamma-producing (Th1-like) cells into the inflamed retina in vivo is preferentially regulated by P-selectin glycoprotein ligand 1:P/E-selectin interactions. J. Immunol..

[62] Xu H., Manivannan A., Liversidge J., Sharp P.F., Forrester J.V., Crane I.J. (2003). Requirements for passage of T lymphocytes across non-inflamed retinal microvessels. J. Neuroimmunol..

[63] Tur-Gracia S., Martinez-Quiles N. (2021). Emerging functions of cytoskeletal proteins in immune diseases. J. Cell Sci..

[64] Pei D. (1999). Leukolysin/MMP25/MT6-MMP: A novel matrix metalloproteinase specifically expressed in the leukocyte lineage. Cell Res..

[65] Kojima S., Itoh Y., Matsumoto S., Masuho Y., Seiki M. (2000). Membrane-type 6 matrix metalloproteinase (MT6-MMP, MMP-25) is the second glycosyl-phosphatidyl inositol (GPI)-anchored MMP. FEBS Lett..

[66] English W.R., Velasco G., Stracke J.O., Knauper V., Murphy G. (2001). Catalytic activities of membrane-type 6 matrix metalloproteinase (MMP25). FEBS Lett..

[67] Lerchenberger M., Uhl B., Stark K., Zuchtriegel G., Eckart A., Miller M., Puhr-Westerheide D., Praetner M., Rehberg M., Khandoga A.G. (2013). Matrix metalloproteinases modulate ameboid-like migration of neutrophils through inflamed interstitial tissue. Blood.

[68] Hofmaier F., Hauck S.M., Amann B., Degroote R.L., Deeg C.A. (2011). Changes in matrix metalloproteinase network in a spontaneous autoimmune uveitis model. Investig. Ophthalmol. Vis. Sci..

[69] Shiryaev S.A., Remacle A.G., Savinov A.Y., Chernov A.V., Cieplak P., Radichev I.A., Williams R., Shiryaeva T.N., Gawlik K., Postnova T.I. (2009). Inflammatory proprotein convertase-matrix metalloproteinase proteolytic pathway in antigen-presenting cells as a step to autoimmune multiple sclerosis. J. Biol. Chem..

[70] Rempe R.G., Hartz A.M.S., Bauer B. (2016). Matrix metalloproteinases in the brain and blood-brain barrier: Versatile breakers and makers. J. Cereb. Blood Flow Metab..

[71] Lindberg R.L., De Groot C.J., Montagne L., Freitag P., van der Valk P., Kappos L., Leppert D. (2001). The expression profile of matrix metalloproteinases (MMPs) and their inhibitors (TIMPs) in lesions and normal appearing white matter of multiple sclerosis. Brain.

[72] Werry H., Gerhards H. (1992). The surgical therapy of equine recurrent uveitis. Tierarztl. Prax..

[73] Bradford M.M. (1976). A rapid and sensitive method for the quantitation of microgram quantities of protein utilizing the principle of protein-dye binding. Anal. Biochem..

[74] Grosche A., Hauser A., Lepper M.F., Mayo R., von Toerne C., Merl-Pham J., Hauck S.M. (2016). The Proteome of Native Adult Muller Glial Cells from Murine Retina. Mol. Cell. Proteomics.

[75] Hauck S.M., Hofmaier F., Dietter J., Swadzba M.E., Blindert M., Amann B., Behler J., Kremmer E., Ueffing M., Deeg C.A. (2012). Label-free LC-MSMS analysis of vitreous from autoimmune uveitis reveals a significant decrease in secreted Wnt signalling inhibitors DKK3 and SFRP2. J. Proteom..

[76] Hauck S.M., Lepper M.F., Hertl M., Sekundo W., Deeg C.A. (2017). Proteome Dynamics in Biobanked Horse Peripheral Blood Derived Lymphocytes (PBL) with Induced Autoimmune Uveitis. Proteomics.

[77] Kramer A., Green J., Pollard J., Tugendreich S. (2014). Causal analysis approaches in Ingenuity Pathway Analysis. Bioinformatics.

[78] Perez-Riverol Y., Csordas A., Bai J., Bernal-Llinares M., Hewapathirana S., Kundu D.J., Inuganti A., Griss J., Mayer G., Eisenacher M. (2019). The PRIDE database and related tools and resources in 2019: Improving support for quantification data. Nucleic Acids Res..

